# Orbital and Lumbosacral Plexiform Neurofibroma with PTPN11 Mutation: A Form of the RASopathy

**DOI:** 10.7759/cureus.62301

**Published:** 2024-06-13

**Authors:** Tian Tian

**Affiliations:** 1 Department of Neurology, The First Affiliated Hospital of Zhengzhou University, Zhengzhou, CHN

**Keywords:** mutation, nf1, rasopathy, ptpn11, lumbosacral plexiform, neurofibroma

## Abstract

RASopathies are a group that encompasses a spectrum of related disorders caused by mutations linked to the RAS/mitogen-activated protein kinase (RAS/MAPK) pathway, including neurofibromatosis type 1 (NF1), Noonan syndrome (NS), neurofibromatosis-Noonan syndrome (NFNS), Noonan syndrome with multiple lentigines (NSML). Neurofibromas, as a hallmark of NF1, are extremely rare in patients with other RASopathies. Here we present a case of a 39-year-old Chinese male displaying orbital neurofibromas and lumbosacral plexiform neurofibromas. Histopathology of a CT-guided biopsy of the mass revealed it to be a neurofibroma. The targeted sequencing analysis did not find any pathogenic sequence alteration in the NF1 or NF2 causative genes in blood lymphocytes and hypertrophic nerve tissue, and no additional signs of NF1 were detected, thereby not meeting the diagnostic criteria for NF1. However, we identified a heterozygous mutation (c.836A>G, p.Y279C) in the *PTPN11* gene, which is one of the key components of the RAS-MAPK signaling pathway and is associated with NS, NFNS, and NSML. Nonetheless, a thorough examination did not reveal any signs of these syndromes in the patient. Consequently, it was inferred that this patient likely falls within the spectrum of the RASopathies. This represents a unique case manifesting as orbital and lumbosacral plexiform neurofibromas carrying a *PTPN11* gene mutation, thereby broadening the phenotype spectrum of PTPN11 mutations. Our results also highlight the overlap between RASopathies. Neurofibromas should be considered indicative of a broader spectrum of disorders resulting from mutations in RASopathies other than NF1.

## Introduction

RASopathies are a group of related disorders, including neurofibromatosis type 1 (NF1), Noonan syndrome (NS), neurofibromatosis-Noonan syndrome (NFNS), Noonan syndrome with multiple lentigines (NSML) arise due to mutations in genes associated with the Ras/mitogen-activated protein kinase (RAS/MAPK) pathway. The RAS-MAPK pathway plays a pivotal role in cell proliferation, mobility, apoptosis, morphological determination, organogenesis, and growth [1,2]. Over the past decade, advances in molecular biology and gene sequencing have highlighted substantial genetic diversity and overlapping phenotypes among various RASopathies [3,4].

Plexiform neurofibromas, as the hallmark of NF1 [5], are infrequently observed in patients with other RASopathies. Here we report a patient displaying orbital neurofibromas and lumbosacral plexiform neurofibromas while carrying a heterozygous mutation (c.836A>G, p.Y279C) in the protein tyrosine phosphatase nonreceptor 11 (PTPN11) gene. PTPN11 is one of the key components of the RAS-MAPK signaling pathway and is linked with NS, NSML, and NFNS [6]. Nonetheless, a comprehensive examination revealed no signs of these syndromes in the patient. We speculated that this patient might belong to the spectrum of the RASopathies. Notably, this represents the first case of multiple neurofibromas, including orbital and lumbosacral plexiform neurofibromas, associated with a PTPN11 mutation, thereby broadening the phenotype spectrum of the PTPN11 mutation.

## Case presentation

A 39-year-old Chinese male experienced a gradual onset of weakness in all four extremities over 6 months. His symptoms indicated a potential peripheral neuropathy and subsequent magnetic resonance imaging (MRI) uncovered bilateral intra-orbital lesions and plexiform neurofibromas in the lumbosacral plexi. The patient was otherwise healthy. No other signs or symptoms of NF-1 or NF-2 were found. His family history was negative for neurofibromatosis or inherited disorders, and he was not taking any medications.

On examination, his height was 181 cm, weight 67.8 kg, and BMI 20.7 kg/m2. His joints were hypermobile. Multiple subcutaneous nodules were palpable in the neck. The later biopsy confirmed neurofibromas, categorized as World Health Organization (WHO) grade I. The skin and mucous membranes were otherwise normal. In particular, no café au lait spots, axillar, or inguinal freckling was found. Ophthalmological assessment disclosed no Lisch nodules; however, an ultrasound detected a hypoechoic mass in the superior temporal quadrant of the right orbit. Hearing was normal.

Neurological examination revealed distal weakness in the left foot [Medical Research Council Scale (MRC) 3- for both toe flexion and extension] and the right arm (MRC 4); symmetrically weak biceps, triceps, and absent ankle reflexes; distal hypesthesia in the left foot; decreased vibration sensation at the left ankle. Electrophysiological testing indicated a severe axonal and demyelinating sensorimotor polyneuropathy. Routine laboratory tests yielded results within normal ranges. Lumber puncture indicated a normal opening pressure, while cerebrospinal fluid (CSF) analysis showed a normal white blood cell count but an elevated protein level of 1488.9 mg/L (reference range 150-450 mg/L).

**Figure 1:**
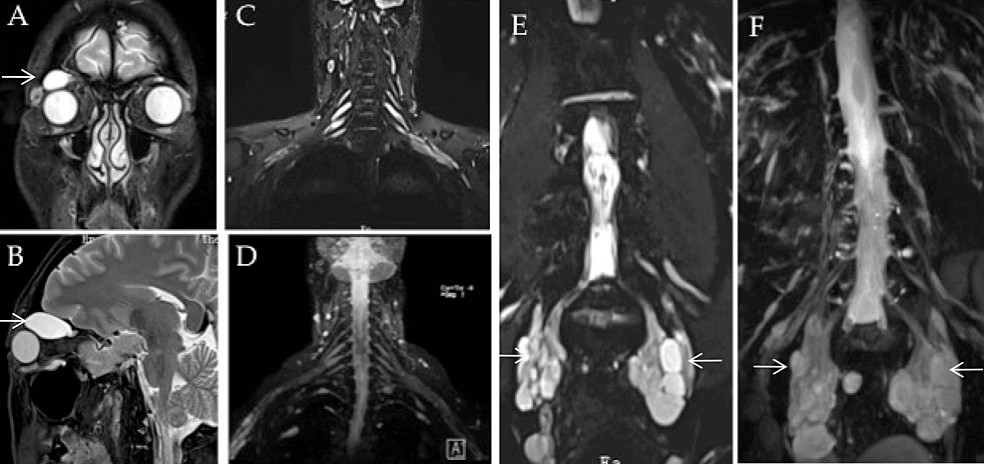
MRI images. T2-weighted MRI images of the orbit show bilateral intraorbitally hyperintense lesions of the ophthalmic division of the fifth cranial nerves (larger on the right side). A mass in the right orbit is located between the osseous roof and the superior orbital muscles. Coronal T2-TIRM-weighted images and reconstructed MIP 3D SPACE images illustrate normal cervical nerve roots and brachial plexus (C, D), and multiloculated tumors involving the lumbosacral plexus with bilateral and symmetric extension to the pelvic region (E, F). The imaging findings were consistent with a plexiform subtype. TIRM: turbo inversion recovery magnitude; MIP: maximum intensity projection; 3D SPACE: three-dimensional sampling perfection with application-optimized contrasts using different flip angle evolution.

MRI of the orbit (Figures 1A-1B) revealed well-defined elongated masses bilaterally within the orbit, involving the ophthalmic division of the fifth cranial nerves. The mass appears larger on the right side. No other cranial nerve tumors were found. T2-TIRM (turbo inversion recovery magnitude)-weighted images and reconstructed maximum intensity projection (MIP) three-dimensional sampling perfection with application-optimized contrasts (3D SPACE using different flip angle evolution) images showed that the cervical nerve roots and brachial plexus were normal (Figures 1C-1D). Furthermore, multiloculated tumors involving the lumbosacral plexus with bilateral and symmetric extension to the pelvic region were observed, consistent with a plexiform subtype (Figures 1E-1F). Spinal MRI indicated scoliosis without additional bone deformities, and both the brain and spinal cord appeared normal. Longitudinal ultrasound images demonstrated multiple hypoechoic fascicles along the bilateral brachial plexuses, lumbosacral plexuses, and femoral nerves, showing varying degrees of enlargement and a tortuous course, indicative of plexiform neurofibromas. Histopathology from a CT-guided biopsy of the lumbosacral plexus tumors confirmed a WHO Grade I neurofibroma. A biopsy of the sural nerve demonstrated a preferential loss of myelinated nerve fibers, with only a small number of thin myelinated nerve fibers left.

**Figure 2:**
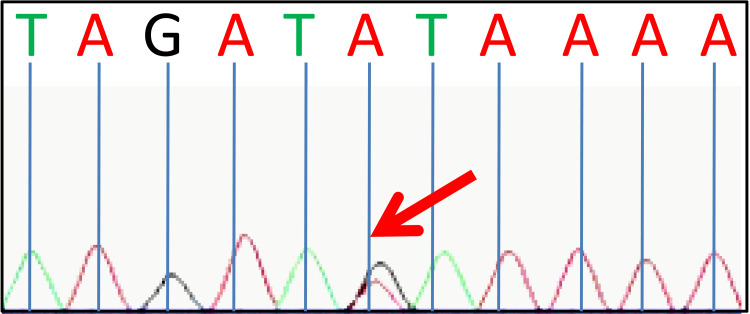
Next-generation sequencing analysis of all RAS-pathway genes identified a heterozygous missense mutation of the PTPN11 gene (c.836A>G, p.Y279C).

Based on the initial suspicion of NF1, an extensive gene screening was conducted. The targeted sequencing analysis did not uncover any pathogenic sequence alteration in the NF1 or NF2 causative genes (NF1, NF2, SMARCB1, or LZTR1) in blood lymphocytes and hypertrophic nerve tissue, and no additional indicators of NF1 were detected, thereby not meeting the diagnostic criteria for NF1. However, a heterozygous germ-line PTPN11 missense mutation (c.836A>G, p.Y279C) was identified (illustrated in Figure 2), a pivotal component of the RAS-MAPK signaling pathway. This mutation was predicted as potentially disease-causing by Mutation Taster and assessed to disrupt protein function by sorting intolerant from tolerant (SIFT). The PTPN11 p.Y279C mutation (c.836A>G) has been previously associated with NS, NSML, and NFNS [6]. Despite this, a thorough examination did not reveal any signs of these syndromes in the patient. Consequently, it was inferred that this patient might be one of the RASopathies.

In view of multilevel involvement, an orthopedic and neurosurgical consult was sought but the patient didn’t undergo surgical intervention owing to the improbability of complete removal.

## Discussion

Spinal nerve tumors have always been considered a characteristic feature of NF1 [3]. The occurrence of plexiform neurofibromas in the absence of NF1 is extremely rare [7]. Nevertheless, no NF1 or NF2 causative gene alteration was identified in this patient. Research suggested that individuals with spinal neurofibromas but few or no other NF1 indicators may be linked to milder NF1 mutations or other genetic causes [8, 9]. As anticipated, a heterozygous mutation in PTPN11 was detected, leading to the conclusion that the patient falls into the category of RASopathies. The sole manifestation observed was multiple neurofibromas, predominantly affecting the orbital and lumbosacral plexiform regions.

Plexiform neurofibromas without other stigmata of NF1 have been described [10,11]. Blakley et al. observed a cohort of patients with multiple biopsy-proven neurofibromas devoid of NF1 features and proposed the term “multiple isolated neurofibromas” to describe this rare condition [12]. Orbital neurofibromas are also rare, constituting 0.6-2.4% of all orbital tumors [13]. They are only infrequently associated with NF (7-10%) [14,15]. Several cases with isolated clinical findings have been reported [16-18]. In a study by Babovic-Vuksanovic et al., four patients with neurofibromas affecting orbital and spinal nerves, along with distinctive facial features and a marfanoid habitus, were described. Interestingly, these patients also presented with scoliosis, joint hypermobility, and a marfanoid habitus, mirroring the symptoms observed in our patient. However, no PTPN11 mutation or other gene mutation was detected. The authors speculated that these cases might represent a new syndrome [5]. Further research is necessary to explore the potential reasons for this difference, and continuous follow-up of the case is warranted. Additionally, the inclusion of more cases is essential for a comprehensive understanding of this potential new syndrome.

The occurrence of isolated neurofibromas in individuals with RASopathies other than NF1 is uncommon [6]. This is the first case featuring both orbital and lumbosacral plexiform neurofibromas carrying a *PTPN11 *gene mutation, thereby broadening the clinical spectrum of PTPN11 mutations. The *PTPN1 *gene encodes the nonreceptor PTP SHP-2, a cytoplasmic protein tyrosine phosphatase that acts as a transducer. SHP-2 affects cell proliferation and differentiation across various cell types and may be implicated in nerve overgrowth and neurofibromas formation in NSML [6].

RASopathies exhibit notable genetic and phenotypic heterogeneity. On the one hand, mutations in the same gene can give rise to different syndromes, as seen in NS and NSML, primarily attributed to PTPN11 mutations. On the other hand, malfunctions in distinct proteins at various levels of the RAS/MAPK cascade can result in overlapping clinical phenotypes. Growing evidence supports the idea that the products of NF1 and *PTPN11 *gene-neurofibromin and SHP-2, respectively, exert their modulatory influence through a shared pathway. Consequently, both NF1 and *PTPN11 *gene mutations are linked to the occurrence of plexiform neurofibroma. Numerous case reports detailing patients with borderline phenotypes underscore this clinical overlap. For instance, a de novo NF1 mutation in a woman previously diagnosed with NSML. A study described three children meeting NF1 clinical criteria but diagnosed with NSML and carrying the *PTPN11 *mutation (p.Thr468Met), the same mutation identified in our patients [19]. When the diagnosis of neurofibroma is uncertain, genetic analysis of RASopathy genes should be considered.

The advancement of individualized cancer treatment holds promise for diverse cancer types through the customization of drugs and treatment plans based on unique individual genomes or epigenome mutations specific to tumor cells. In the realm of gene therapy, the revolutionary CRISPR/Cas9 technology has emerged as a potent tool, enabling precise and targeted modification of the DNA sequence of cells. This capability can rectify genetic defects and pave the way for innovative therapeutic modalities. Numerous clinical studies have delved into the gene editing functionality of the CRISPR/Cas9 system and demonstrated its favorable strides in the management of glioblastoma (GBM) patients [20]. Hence, in the foreseeable future, patients grappling with neurofibroma might also stand to gain from gene editing.

## Conclusions

This is the first case characterized solely by the presence of orbital and lumbosacral plexiform neurofibroma with a PTPN11 mutation, thereby extending the phenotype associated with PTPN11 mutations. Our results highlight the overlap between RASopathies. Isolated neurofibromas should be regarded as indicative of a broader spectrum of disorders resulting from mutations in RASopathies other than NF1.
